# Choline During Pregnancy and Child Neurodevelopment: A Systematic Review of Randomized Controlled Trials and Observational Studies

**DOI:** 10.3390/nu17050886

**Published:** 2025-02-28

**Authors:** Jacqueline F. Gould, Sonia Hines, Karen P. Best, Luke E. Grzeskowiak, Olivia Jansen, Tim J. Green

**Affiliations:** 1 Women and Kids Theme, South Australian Health and Medical Research Institute, Adelaide, SA 5006, Australia; jacqueline.gould@sahmri.com (J.F.G.); karen.best@sahmri.com (K.P.B.); luke.grzeskowiak@flinders.edu.au (L.E.G.); olivia.jansen@sahmri.com (O.J.); 2 School of Psychology, Faculty of Health and Medical Sciences, The University of Adelaide, Adelaide, SA 5005, Australia; 3 Flinders University Rural and Remote Health SA, Flinders University, Alice Springs, NT 0870, Australia; sonia.hines@flinders.edu.au; 4 Mparntwe Centre for Evidence in Health, Flinders University: A JBI Centre of Excellence, Alice Springs, NT 0870, Australia; 5 Adelaide Medical School, Faculty of Health and Medical Sciences, The University of Adelaide, Adelaide, SA 5005, Australia; 6 Flinders Health and Medical Research Institute, College of Medicine and Public Health, Flinders University, Adelaide, SA 5042, Australia; 7 College of Nursing and Health Sciences, Flinders University, Adelaide, SA 5042, Australia

**Keywords:** choline, pregnancy, prenatal, child neurodevelopment, supplementation, systematic review

## Abstract

Background: Most pregnant women have choline intakes below recommendations. Animal studies suggest that choline supplementation during pregnancy improves cognitive outcomes in the offspring. This review aims to determine whether higher choline levels during pregnancy are associated with improved child brain development. Methods: We systematically reviewed the evidence for the role of choline in pregnancy for human neurodevelopment in clinical trials and observational studies. Results: We identified four randomized trials of choline supplementation in pregnancy and five observational studies of prenatal choline. Neurodevelopmental assessments of these studies were reported across 20 eligible publications. Within both the trials and observational studies, most neurodevelopmental outcomes assessed did not support the hypothesis that higher prenatal choline benefits neurodevelopment. Among identified clinical trials, there were some instances where children whose mothers received choline supplementation had a better score on a neurodevelopmental measure. Still, each trial included multiple outcomes, and most were null. Observational studies were mixed as to whether an association between prenatal choline and an aspect of child neurodevelopment was identified. Critical limitations were present across clinical trials and observational studies, preventing confidence in the results and evidence base. Conclusions: Current evidence is insufficient to support or refute the hypothesis that increasing choline intake in pregnancy improves the neurodevelopmental outcomes of the child.

## 1. Introduction

Maternal nutrition during pregnancy is an important determinant of infant health and development. Choline is an essential nutrient necessary to facilitate early brain development. Choline is required for the synthesis of the neurotransmitter acetylcholine and betaine (a vital methyl donor), as well as in osmoregulation. Choline is also a component of phosphatidylcholine, a phospholipid found in high amounts in neural tissue [1] and is involved in a broad range of critical physiological functions across all life stages [2]. Choline is synthesized endogenously from phosphatidylcholine, but endogenous choline synthesis is insufficient to meet needs, especially during periods of rapid growth [1,2], such as in pregnancy when the fetal brain accrues large amounts of phosphatidylcholine [3].

Choline was not officially recognized as an essential nutrient by the U.S. Institute of Medicine until 1997, when an Adequate Intake (AI) for choline was set at 450 mg/d during pregnancy [1]. In 2005, the Australian National Health and Medical Research Council adopted the 1997 U.S. AI for choline but set it 10 mg lower at 440 mg/d in pregnancy. Although multivitamins are becoming increasingly popular during pregnancy, choline is absent from most prenatal supplements. Animal-source foods (particularly chicken eggs) are the main contributor to dietary choline intake in high-income countries [4,5,6,7]. Most surveys of pregnant people in high-income countries show that choline intakes are well below the AI [5,8,9,10,11,12]. For example, in the U.S. NHANES cohort (2001–2014), the mean choline intake among pregnant women was ~320 mg/d, with <10% exceeding the AI [9,10]. The average choline intake in Australia was only 251 mg/d, and less than 1% of pregnant women exceeded the AI in the 2011–2012 National Nutrition and Physical Activity Survey [9,10]. There is growing interest in whether increasing maternal choline intake improves neurodevelopmental outcomes in the child [3,8,13,14,15,16,17,18]. Several animal studies suggest that choline supplementation during pregnancy improves cognitive outcomes in the offspring [13,19]; however, the effect on humans is unclear [8,13,14,15,16]. A handful of human observational studies and trials of choline in pregnancy have recently emerged. Here, we aim to systematically review the evidence from cohort studies and trials to determine whether higher prenatal choline intake leads to better neurodevelopmental outcomes for children.

## 2. Materials and Methods

We conducted our systematic review according to the reporting guidelines outlined in the Preferred Reporting Items for Systematic Reviews and Meta-Analysis [20]. This systematic review is registered on the PROSPERO registry (https://www.crd.york.ac.uk/prospero/display_record.php?ID=CRD42020206010, registered on 6 October 2020, last edited on 10 May 2022, last accessed on 26 February 2024).

Published articles were eligible for inclusion in this review if they investigated the role of dietary maternal choline intake (including phosphatidylcholine, betaine, sphingomyelins, or eggs) in pregnancy with an assessment of child neurodevelopment. We were primarily interested in trials but also included observational studies, which we considered separately. We included trials if the intervention included choline alone, without other nutrients. We also included observational studies if the exposure was prenatal dietary intake of choline (including from supplement use) and/or prenatal circulating choline concentrations, a biomarker of choline intake. Outcomes eligible for inclusion in the review were any measure of neurodevelopment conducted during infancy or childhood, such as cognitive functioning or behavior. The population of interest during the intervention or exposure was pregnant women, and participants with the outcome of interest were children. We excluded other study designs (such as reviews), animal studies, human trials, or observational studies where the intervention or exposure was postnatal or later in childhood. Manuscripts not published in English were omitted.

We searched PubMed, EMBASE (Elsevier), PsycINFO (Ovid), and The Cochrane Library for eligible articles, with search alerts to capture new potentially eligible publications for inclusion until March 2024. Search terms are available upon request. Search results were imported into COVIDENCE, and duplicates were removed. A research assistant and one author (TJG) cross-checked the removed duplicates for potential errors and screened search results for eligibility. Reference lists of eligible articles and any similar reviews were also screened for relevant manuscripts.

The included studies were independently reviewed by two authors (TJG and JFG), who extracted data on general information (authors, year, study location, and country), study characteristics (study design, sample size, choline status, and type and timing of intervention), and neurodevelopment outcomes. Results from the two authors were reviewed and compared to determine and discuss differences, with a third author (LEG) available to review disagreements, if needed. Data were extracted using a standardized, pre-prepared extraction form. We noted limitations and possible biases, such as a small sample, suboptimal exposure or outcome measures, or other indications of poor study quality (for example, inadequate consideration of confounders). Cochrane’s risk of bias tool was used to evaluate the risk of bias (including selection, performance, detection, attrition, reporting, and other possible sources of bias) in trials. For observational and case–control studies, two authors (KPB, and OJ) assessed quality using the Newcastle–Ottawa Scale (NOS) tool for evaluating the quality of non-randomized studies across three categories: Selection, Comparability, and Outcome [21]. Results from the two authors were compared to determine and discuss differences, with a third author available to review disagreements (JFG) [22]. The NOS assigns a maximum sum score of 9 for both case–control and cohort studies, with higher scores indicating higher study quality, and it is endorsed by the Cochrane Collaboration.

## 3. Results

**Figure 1:**
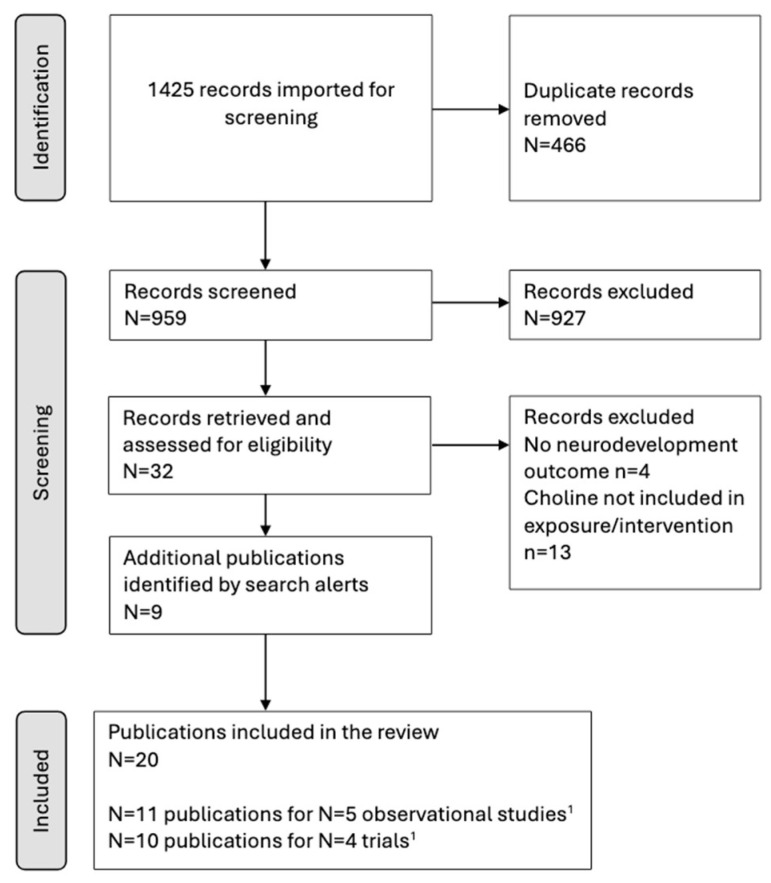
Flow of trials and observational study publications through the literature search and screening for eligibility. ^1^ One publication reported additional analyses of a cohort study and a trial.

The search returned 1425 records, with 959 remaining after duplicates were removed (Figure 1). Of these, 927 were excluded after the abstracts were reviewed because they did not meet the inclusion criteria. The full texts of 32 records from the initial search were examined in detail, of which 21 were excluded (including a prenatal trial of multivitamins that contained choline [23,24]), and ongoing search alerts were screened.

A total of 20 included records pertained to four individual trials [25,26,27,28] (with neurodevelopmental outcomes published across 10 peer-reviewed publications [11,12,29,30,31,32,33,34,35,36]) and five observational studies [11,29,30,31,36] (with outcomes published in 7 full-text publications [25,26,27,28,37,38,39] and 2 conference abstracts [40,41]). There was one record [42] where authors combined and re-analyzed select data from an included cohort [31] with one of the included trials [26].

Trials of prenatal choline were heterogeneous in terms of the form and dose of choline provided, as well as the intervention period and outcome timing and assessment. Observational studies likewise varied in methodology, including measurement (timing, method, and definition) of exposure and outcome. Populations in both study designs were most often from high-income countries, and sample sizes were typically small. Results and conclusions were mixed but largely null.

### 3.1. Participants

#### 3.1.1. Clinical Trials

The four trials [25,26,27,28] were conducted on 338 participants and ranged in size from 29 to 140 participants (see Table 1). One trial [37] had an additional follow-up assessment [26] and subgroup analyses [42] published, one trial [28] had one follow-up measurement published [39], and another trial [27] had follow-up assessment results published [37] and available in abstracts only [40,41], but these were included here. Three trials were conducted in the U.S., all on healthy women. The other trial was conducted on pregnant women in South Africa who were described as heavy drinkers. The trial aimed to mitigate the adverse effects of prenatal alcohol exposure [28,39], based on animal models indicating choline may reduce the impact of alcohol [43].

#### 3.1.2. Observational Studies

The five observational studies [11,29,30,31,36] included a total of 3192 participants but ranged in size from 154 [12] to 2128 [30] participants (see Table 2). The manuscript by Boeke et al. [12] was a follow-up of participants in Villamor et al. [30]. The results of one observational study [31] were reported across six publications [31,32,33,34,35,42]. All five studies were conducted in high-income countries, and three specified their population consisted of healthy pregnant women. One study was described as a population in which 95% of women had identifiable risk factors for growth restriction [11]. Three studies excluded non-singleton pregnancies or restricted their analysis to singleton births [11,29,30], while two studies did not specify the inclusion or exclusion of multiple births [31,36]. Other exclusion criteria included inability to answer questions in English and women >22 weeks’ gestation [30], increased risk of pre-term birth [11], fetal anomaly, severe intrauterine growth restriction, corticosteroid use, maternal allergic disease [31], or non-full-term infants [11].

### 3.2. Exposure or Intervention

#### 3.2.1. Clinical Trials

Of the trials, two gave choline as phosphatidylcholine [25,26] and the other two as choline salts, either chloride [27] or bitartrate [28]. Three trials included a placebo [25,26,28], two used corn oil as the placebo [25,26], and the other did not specify [28]. Caudill et al. did not have a placebo [27] but used two doses of choline 480 or 930 mg/d choline. The supplementation period varied greatly; two trials continued supplementation until delivery; one started in the third week of pregnancy [27]; and the other midway in the second trimester [28]. Two trials began in the second trimester and continued to 90 days postpartum, one supplementing the mother postpartum [25], and the other supplementing the infant directly [26]. Maternal doses of choline ranged from 480 [27] to 2000 mg/d [28].

#### 3.2.2. Observational Studies

Four observational studies used plasma or serum choline to measure status in pregnancy (see Table 2). Signore et al. [29] measured serum total and free choline multiple times during pregnancy and in cord blood, and Wu et al. [11] measured total plasma choline at 16 and 36 weeks’ gestation. Freedman et al. [31] measured serum choline at 16 weeks’ gestation but some papers also reported measures taken at 28 weeks’ of pregnancy [34,35], or reported measuring choline and metabolite betaine [33,34,35]. Most studies explored choline as a continuous variable, but four publications from the one cohort instead compared choline status categorized as low versus high, where high choline concentration was >7 mM [42], >7.07 mM [34,35], or ≥7.5 mM [32]. Villamor et al. [30] assessed choline intake during the first and second trimesters using a 166-item semi-quantitative Food Frequency Questionnaire that is widely used in large U.S. epidemiological studies and calibrated for use in pregnancy. Irvine et al. administered a 24 h Food Behavior Questionnaire [36].

### 3.3. Neurodevelopmental Outcomes

A wide variety of tests were used to assess differing neurodevelopmental domains at a range of ages in early childhood. All observational studies administered validated, age-standardized tests. In contrast, most of the tests in the trials are experimental and have not been well validated. The heterogeneity of tests, neurodevelopmental domains assessed, and age ranges assessed prevent any meaningful combination or meta-analysis.

#### 3.3.1. Clinical Trials

Within the trials, the parent-rated MacArthur-Bates Short Form Vocabulary Checklist (MCDI) and the Mullen Scales of Early Learning (MSEL) were used by Cheatham et al. at 10 and 12 months of age [25]. The CBCL was completed by parents of children at 40 months of age in Ross et al.’s follow-up [26,37]. The MCDI measures vocabulary abilities, and the authors only analyzed the spoken word scores [25], reflective of receptive abilities. The MESL includes scores for language, visual reception (comprehension of visual information, such as pictures), and motor abilities. Warton et al. 2021 [39] conducted structural magnetic resonance imaging when infants were aged between 1 and 7 weeks postpartum and were sleeping, but not sedated, and regional volumes were extracted for comparison.

The remaining tests applied in trials were experimental assessments that have not been standardized or validated in terms of their administration, scoring, or interpretation of performance, and will be considered with caution. The Fagan Test of Infant Intelligence consists of showing pairs of images to infants and observing whether the infant recognizes an image they have seen previously [28]. This same trial also developed a measure of eye-blink conditioning, in which they attempted to train infants to associate a sound (auditory stimulus) with a puff of air against their eye under the Pavlovian paradigm, and then measured blinking when only the auditory stimulus was present [28]. Cheatham et al. [25] administered a Short-term Visuospatial Memory Task and a Long-term Episodic Memory Task at 10 and at 12 months of age. The Short-term Visuospatial Memory Task involved up to 24 trials per infant, and performance was scored as the percentage of correct responses out of all attempted responses; however, details of the trials were not included. At the second time point, infant response attempts were so low that only data from the first assessment were compared between groups [25]. The Long-term Episodic Memory Task involved four trials, although details of the trials were again not reported [25]. Electrophysiological recordings were conducted at a mean age of 33 days and again at a mean age of 89 days to determine inhibition of the P50 component of the auditory cerebral evoked responses to paired sounds [26]. The primary outcome of the measure was the P50 inhibition ratio, defined as the amplitude of the P50 response across repeated stimuli, where a smaller ratio indicates inhibition on repeated stimuli. The authors categorized P50 ratios as normal and abnormal [26]. Latencies and amplitudes of the individual P50 responses were also extracted and compared, although there are no details of the stimuli or the methodology of the electrophysiological recordings [26]. A Visual Attention Task was administered at 4, 7, 10, and 13 months of age to measure eye movements in reaction to visual stimuli on a display screen [27]. The primary outcome of this assessment and this trial was the mean reaction time for eye movements to a presented stimulus; additionally, the number of eye movements made in anticipation of the stimulus was also analyzed [27]. A later follow-up of these children, as they reached 7 years of age, involved a Sustained Attention Task of cognitive control of voluntary attention [38]. For this task, participants were seated in front of a screen and asked to rapidly indicate whether they saw a gray square on a gray background across 216 trials. This trial also has the results of two other tests conducted at the 7-year follow-up presented in published abstracts [40,41]. However, it is difficult to discern if there are other tests and outcomes that are not yet available. In one abstract, authors report an executive functioning test, the Tower of London Task, which requires children to move rings between pegs into a certain order with the minimum number of moves [40]. Outcomes were the total score across all attempts, the number of correct moves in the first attempt, and the planning time of the first move. The authors of the other abstract reported a computerized Color–Location Memory Task where children had to recall the location of dots on a carton after a delay, with increasing complexity due to the length of delay and a number of dots to memorize [41].

#### 3.3.2. Observational Studies

Among the observational studies, the Wechsler Preschool and Primary Scale of Intelligence (WPPSI) was used to measure intelligence quotient (IQ) at 3–4 years of age (WPPSI-IV) [36] and at age 5 in the largest of the observational studies (WPPSI-R) [29]. Villamor et al. used the Peabody Picture Vocabulary Test (PPVT) for receptive (comprehension) language skills and the Wide Range Assessment of Visual Motor Abilities (WRAVMA) in children at age 3 [30]. A subset of these children was tested again at age 7 [12], using the Wide Range Assessment of Memory and Learning-Second Edition (WRAML2) to measure design memory and picture memory and the Kaufman Brief Intelligence Test-Second Edition (KBIT2). Wu et al. [11], used the Bayley Scales of Infant Development-Third Edition (Bayley-III) to assess cognition, language, and motor abilities at 18 months of age, the most established test to establish neurodevelopment in children less than 42 months. Irvine et al.’s [36] 3–4-year follow-up also included the NEPSY-II (A Developmental Neuropsychological Assessment), as well as the Dimensional Change Card Sort (DCCS), Spatial Span, and Boy–Girl Stroop to assess aspects of executive functioning. Investigators also included a measure of motor abilities, the Movement Assessment Battery for Children (MABC) to measure motor skills [36]. One observational study measured cerebral inhibition at 1 month of age via auditory evoked potential P50 during sleep and reported results in three papers [31,33,42]. Then, when infants reached 3 months, parents completed the IBQ-R, Infant Behavior Questionnaire-Revised Short Form (IBQ-R), which was reported across four papers [31,32,33,42]. The focus of one of these papers was the interaction between maternal infection and choline in pregnancy [31], and the authors published a separate paper focusing on the subgroup of mothers who experienced viral respiratory infections [32]. Another paper from this cohort targeted the interaction effect of choline and maternal marijuana use in pregnancy [33]. A later follow-up of these children at 18, 30, 40, and 48 months included the Child Behavior Checklist (CBCL), where scores were averaged across all time points [34], and a WPPSI-IV at 4 years [35].

**Table 1:** Summary of trials of choline exposure in pregnancy and child neurodevelopment.

Author/Setting	Participants	Interventions	Outcome Measure and Follow-Up	Results
Cheatham et al. 2012 [25] Chapel Hill NC, U.S.	N:140 Healthy	Period:18 wk. of gestation to 90 d postpartum Treatment: 750 mg/d choline as phosphatidylcholine or placebo (corn oil)	Age: 10 and 12 mo. Outcome: MCDI-SF; MSEL; Short-term Visuospatial Memory Task; Long-term Episodic Memory Task	No effect of treatment on any outcome
Ross et al. 2013 [26] Denver CO, U.S.	N:100 Healthy	Period: Second trimester to 70–90 d postpartum Treatment: 900 mg/d choline as phosphatidylcholine or placebo (corn oil). Infants 100 mg/d phosphatidylcholine or placebo in an oral suspension.	Age: 5 and 13 wk. Primary Outcome: normal or abnormal P50 inhibition ratio according to electrophysiological recordings of cerebral inhibition Outcome: latencies and amplitudes of the individual P50 responses according to electrophysiological recordings of cerebral inhibition Age: 6 mo. Outcome: MSEL	Infants whose mothers received choline versus placebo were more likely to have normal inhibition at 5 wks (beneficial effect). No difference at 13 wks. No effect on MSEL
Ross et al. 2016 [37]	N:49		Age: 40 mo. Outcome: CBCL-parent report	Fewer attention problems were reported and less social withdrawal in choline versus the placebo group. Aggression, emotionality, anxiety/depression, sleep, somatic, internalizing, externalizing, and total scores did not differ.
Hunter et al. 2021 [42]	N:15 Black American mothers			Infants whose mothers received choline versus placebo were more likely to have normal P50 inhibition (beneficial effect, unclear whether 5 week and/or 13-wk assessment). Males of mothers who received choline had lower CBCL withdrawn and attention problem scores, but no other differences and no differences among females.
Caudill et al. 2018 [27] Ithaca NY, U.S.	N:29 Healthy	Period: third wk. of gestation until delivery Treatment: 480 or 930 mg/d choline chloride	Age: 4, 7, 10, and 13 mo. Primary outcome: mean reaction time for eye shifts according to a Visual Attention Task Outcome: Number of predictive eye movements (made in anticipation of a stimulus presenting) according to a Visual-Attention Task.	Reaction time averaged across the four ages was significantly faster for infants born to mothers in the 930 vs. 480 mg/d choline group. The number of anticipatory eye movements did not differ between groups.
Nevins et al. 2018 [40]	N:20		Age: 7 y Outcome: Tower of London task-total score, correct moves, and planning time	The total score did not differ between choline groups, nor did planning time. Children born to mothers in the 930 vs. 480 mg/d choline group had more correct moves at the first attempt.
Bahnfleth et al. 2019 [41]	N:20		Age: 7 y Outcome: Color Location Memory Task	Children whose mothers were in the 930 vs. 480 mg/d choline group passed more levels of increasing difficulty.
Bahnfleth et al. 2022 [38]	N:20		Age: 7 y Outcome: Sustained Attention Task	A total of 930 versus 480 mg/d choline group had a better overall score as well as more correct responses.
Jacobson et al. 2018 [28] Cape Town, South Africa	N: 69 heavy drinkers	Period: by 23 wk. of gestation until delivery Treatment: 2000 mg/d of choline bitartrate or placebo (not defined)	Age: 6.5 and 12 mo. Outcomes: eyeblink conditioning, Fagan Test of Infant Intelligence (visual recognition memory)	At 6.5 mo., the choline group was more likely to be classified as meeting the criterion for eyeblink conditioning. At 12 but not 6.5 mo. the choline group had faster recognition memory processing speed. No difference in recognition processing speed
Warton et al. 2021 [39]	N: 67		Age: 1–7 wk. Outcomes: Regional brain volumes were measured using structural magnetic resonance imaging. Subcortical regions were manually segmented. All volumes were adjusted for age and total intracranial volume.	Six of the twelve regions in the brain were larger in the choline than in the placebo group. Larger right putamen and corpus callosum were related to higher Fagan scores at 12 mo. An NS trend toward partial mediation of the choline effect on recognition memory.

Fagan, Fagan Test of Infant Intelligence; MCDI-SF, Mac- Arthur-Bates Short Form Vocabulary Checklist: Level I; MSEL, Mullen Scales of Early Learning; CBCL, Child Behavior Checklist.

**Table 2:** Summary of observational studies of choline exposure in pregnancy and child neurodevelopment.

Author	Participants	Exposure	Follow-Up	Confounder Adjustments	Results
Signore et al. 2008 [29] AB, U.S. Infant Growth Project cohort	N:400 Healthy women receiving care from the public health department	Period: 16–18, 24–26, 30–32, and 36–38 wk pregnancy and cord. Measure: Serum total and free choline.	Age: 5 y Outcomes: WPPSI-R	Maternal PPVT-R raw score, HSQ score, poverty status, maternal race, education level, smoking, alcohol intake, gestational age at delivery, and infant sex.	No association between maternal or cord serum total and free choline at any time point for any neurodevelopmental outcome.
Villamor et al. 2012 [30] MA, U.S. Project Viva cohort	N:2128 Healthy women receiving care at a large practice	Period: first and second trimester Measure: Choline intake measured by FFQ	Age: 3 y Outcomes: PPVT-III, WRAVMA	Maternal ethnicity, age, parity, smoking, body mass index, PPVT-III, education, energy intake, fish and iron intake; paternal education; household income, infant sex, and primary language.	No association between choline intake and neurodevelopmental outcomes.
Boeke et al. 2013 [12]	N:895		Age: 7 y Outcomes: WRAML-2, KBIT-2		Top quartile choline intake in the second trimester was associated with modestly better WRAML (child visual memory) at 7 y. No other associations.
Wu et al. 2012 [11] Vancouver, Canada	N:154 Healthy	Period: 16, and 36 wk. of pregnancy. Measure: Plasma choline	Age: 18 mo. Outcome: Bayley-III	Maternal IQ, ethnicity, age phosphatidylethanolamine docosahexaenoic acid, infant sex, and breastfeeding duration.	A positive association was found between better infant cognitive test scores and higher maternal plasma choline at 16 wks.
Freedman et al. 2019 [31] Colorado, U.S.	N: 201 Healthy	Period: 16 wk. of pregnancy. Measure: Serum choline	Age: 1 mo. Outcome: cerebral auditory evoked potential as P50 amplitude Age: 3 mo. Outcome: IBQ-R	The primary analyses explored whether choline lessens any detrimental effects of maternal infection in pregnancy. Maternal age, education obesity, depression, and infant sex.	A significant interaction between choline status and infection where choline levels were associated with lower P50 amplitudes (beneficial effect) among infants whose mothers had experienced an infection. Similarly, choline was associated with increased IBQ-R Regulation, but not Surgency or Negativity for infants whose mothers were infected.
Freedman et al. 2020 [32]	N:89 Women who experienced a viral respiratory infection or no infections during pregnancy		Age: 3 mo. Outcome: IBQ-R	The primary analyses explored whether choline status ≥7.5 mM lessens any detrimental effects of maternal viral respiratory infection in pregnancy. Maternal age, education, obesity, anxiety, depression, and infant sex.	A significant interaction between choline and viral respiratory infection, where children whose mothers had choline status ^3^7.5 mM had higher regulation scores, particularly within regulation the subscale of attention if their mother had experienced infection. The effect of other regulation subscales, nor surgency or negativity were reported.
Hoffman et al. 2020 [33]	N: 201	Period: 16 wk. of pregnancy. Measure: plasma choline and metabolite betaine	Age: 1 mo. Outcome: cerebral auditory evoked potential as P50 amplitude Age: 3 mo. Outcome: IBQ-R	The primary analyses explored whether choline lessens any detrimental effects of prenatal marijuana use. Maternal health, sociodemographic, prenatal and delivery characteristics, and infant sex.	A significant interaction between choline status and marijuana use where choline levels were associated with lower P50 amplitudes (beneficial effect) among infants whose mothers had used marijuana during pregnancy. Similarly, choline was associated with increased IBQ-R Regulation, but not surgency or negativity for infants whose mothers used marijuana.
Hunter et al. 2021 [42]	N: 149 N:23 Black Americans N:126 White Americans			The primary analyses explored whether choline influences the risk of predisposition to mental health for black Americans.	Higher choline at 16 weeks but not 28 weeks, was inversely associated with lower P50 (beneficial effect) in infants with Black American mothers. Similarly, higher choline was associated with better IBQ-R regulation scores among infants of Black American women. Choline >7 mM at 16 weeks was associated with better regulation scores in all women, particularly if they had experienced infection in pregnancy.
Hunter et al. 2022 [34]	N: 82	Period: 16 and 28 wk. of pregnancy. Measure: plasma choline and metabolite betaine	Age: 18, 30, 40, 48 mo. Outcome: CBCL (average scores across all time points)	Maternal age, prenatal cannabis use, prenatal infections, lifetime depressive disorder, gestational age at birth, and infant sex.	Maternal choline concentrations >7.07 mM were associated with lower attention problem scores, fewer sleep problems (beneficial effect), and lower withdrawn scores among males only. Choline was associated with attention scores, particularly among children whose mothers had used cannabis or had an infection during pregnancy.
Hunter et al. 2021 [35]	N: 48		Age: 4 y Outcome: WPPSI-IV	Maternal age, pre-pregnancy body mass index, depression, gestational age at birth, birth weight, child age at follow-up assessment, sex, and biological father in the household.	The Processing Speed Score was positively associated with choline concentration >7.07 mM, but no other WPPSI scores were.
Irvine et al. 2023 [36] Alberta, Canada APrON study cohort	N: 309 Metropolitan pregnant women >16 years	Period: 2nd or 3rd trimester Measure: Choline intake measured by 24 hr FFQ	Age: 3–4 y Outcomes: WPPSI-IV, NEPSY-II, MABC-2, Spatial Span, Boy-Girl Stroop, DCCS	Maternal pre-pregnancy body mass index, age, education, income, ethnicity, parity, delivery mode, infant birth weight, sex, and age at assessment, and prenatal serum omega-3, iron, vitamin B12, magnesium, copper, zinc, and selenium status. The interaction with maternal folate status was also explored.	No associations were found for choline intake with all but the DCCS where high choline intake (223 mg/day) and high folate status were associated with lower odds of children receiving a passing score on the DCCS.

CBCL, Child Behavior Checklist; DCCS, Dimensional Change Card Sort; FFQ, Food Frequency Questionnaire; HSQ, Home Screening Questionnaire; IBQ-R, Infant Behavior Questionnaire-Revised Short Form; IQ, Intelligence Quotient; KBIT-2, Kaufman Brief Intelligence Test; MABC, Movement Assessment Battery for Children; NEPSY, A Developmental Neuropsychological Assessment; PPVT, R, revised; The Peabody Picture Vocabulary Test; U.S., United States; WPPSI, Wechsler Preschool and Primary Scale of Intelligence; WRAML, Wide Range Assessment of Memory and Learning; WRAVMA, Wide Range Assessment of Visual Motor Abilities.

### 3.4. Quality and Risk of Bias

#### 3.4.1. Clinical Trials

The quality of the trials somewhat varied (Table 3). The method used to generate the randomization sequence was detailed and deemed adequate to prevent selection bias in three [25,27,28] of the four trials, but may have conferred a high risk of bias for one trial [26]. Allocation concealment and blinding of participants was deemed adequate in three trials and unclear in one trial [26]. Blinding of outcome assessors was not mentioned explicitly in any trial. Only two [27,28] trials had a greater than an 80% follow-up for the primary assessment and were therefore likely to have a low risk of attrition bias; however, follow-up rates for these trials, when children are assessed at older ages, ranged from 50% to 75%. One trial reported post-randomization exclusion of participants, which may have introduced further attrition bias [26].

Selective reporting bias was evident across most trials, and multiple outcomes were selectively included across multiple reports. Data from an assessment were only analyzed at one of the two ages at which they were administered [25]. In another trial manuscript, authors report that participants underwent a 2-day cognitive testing protocol but only provide results of three short assessments, mainly only in abstracts which are not peer-reviewed [38,40,41]. In one trial, the results of an assessment at 12 months were reported in one manuscript [28] and then again in another manuscript that also included results of magnetic resonance imaging measures [39].

There were some instances of other possible bias; for example, only two trials specified a primary outcome of interest [26,27], and none appeared to have conducted a sample size or power calculation. Many of the outcome assessments applied were experimental and open to interpretation and several measures, including two assessments within one trial, with either not described at all or poorly described by study authors [25,26].

#### 3.4.2. Observational Studies

According to the risk-of-bias analysis, most studies were of moderate to high quality with NOS scores ranging from 5 to 8 (Table 4). Most studies scored well in the selection category, as they involved well-defined cohorts and used reliable methods to ascertain exposure, such as biochemical assays for measuring choline levels. However, the reliance on self-reported dietary intake in some studies, such as those by Boeke et al. [12], Villamor et al. [30], and Irvine et al. [36], may introduce recall bias. Several studies were limited by their follow-up rates, which were relatively low and could introduce attrition bias. Comparability was adequate across studies, with many controlling for multiple potential confounders, including maternal age, socioeconomic status, education, and other relevant factors, through multivariable regression analyses. However, some missed key confounders. For example, Wu et al. [11] adjusted for maternal IQ (a strong predictor of child IQ) but not gestational age at birth or parental education. Other studies did not mention adjustment for maternal IQ or other covariates related to the neurodevelopmental outcome of interest [33,34,35,36,42]. Outcome assessment was robust in studies that utilized standardized and validated tools for measuring child neurodevelopment, such as the WPPSI-IV and CBCL. Although some studies did not mention blinding of outcome assessments [11,12,30], this could introduce detection bias. There was evidence of selective reporting bias within one cohort [31], where authors conducted multiple exposure and developmental assessments and published aspects of these across multiple papers where only partial exposure measures or outcomes were reported. Overall, while most studies demonstrated adequate methodological rigor, the potential for selection, detection, and attrition biases should be considered when interpreting the findings [11,29,30,31,36].

### 3.5. Efficacy

#### 3.5.1. Clinical Trials

Within the trials, three [26,27,28] out of the four [25] reported better performance on one or more outcomes of the assessments administered when the children were born to mothers who received a higher dose of choline supplementation, compared with children whose mothers received a placebo or a lower dose of choline. However, in the three trials that reported a benefit, there were 13 different assessments reported, most of which had at least three outcome scores that were compared between intervention groups without suggesting any differences [26,27,28]. The largest trial, which included a placebo group and had a 70% follow-up rate, compared multiple outcomes, and found no differences between the groups [25]. Within the specific domains assessed, there were benefits found to some outcomes from measures of behavior [37,42], brain volume [39], inhibition [26,42], visual attention [27], and executive functioning [28,38,40,41] but no effects were found on measures of language [25,26].

#### 3.5.2. Observational Studies

The two largest observational studies reported no association between choline and any neuro-developmental outcome assessed [29,30], but smaller studies had mixed findings. The largest study to include a measure of intelligence (the KBIT 7 years) found no association with choline intakes in the first trimester [12]. Intelligence assessed with the WPPSI at 5 years [29] or 3-to-4 years of age [35,36] similarly was not associated with choline intake in the second or third trimester [36], or with serum choline measured across pregnancy [29]. In one of these samples, the Processing Speed Score (one of the six composite scores from the WPPSI) was positively associated with maternal choline concentration >7.07 mM [35]. One of the smaller studies (n = 154) found a positive association between the Bayley-III and plasma choline at 16 weeks’ gestation, but did not report associations between the Bayley-III and maternal choline intake measured twice during pregnancy [11].

Assessments of higher-order cognitive abilities, such as executive functioning, were included in two studies. There was no association between choline intake in the second and third trimesters and NEPSY, Spatial Span, Boy–Girl Stroop, or the DCCS at 3 to 4 years of age [36]. The authors reported an interaction where mothers with high choline intake, together with high folate status, were associated with lower odds of children receiving a passing the DCCS. However, the high intake in the sample was 223 mg/day, which was only half of the recommended dietary intake [36]. Children of mothers with a choline intake in the highest quartile in the second trimester scored 1.4 points higher on the WRAML at 7 years, which is a moderately large (approximately 1/3 of a standard deviation) difference in this population [12]. However, no association was observed between choline intakes in the first trimester and WRAML [12].

Of the observational studies that assessed language, the largest again found no association between first- and second-trimester choline intake assessed by FFQ and language (PPVT) at 3 years [30]. Motor abilities were assessed at 3 to 4 years of age in two studies, where neither found an association between prenatal choline intake and performance on the WRAVMA [30] or the MABC [36].

Assessments of behavior included parent ratings and infant cerebral auditory-evoked response inhibition as a biomarker of later neurological circuits responsible for inhibition and attention control (and hence have a role in disorders such as Attention Deficit Hyperactivity Disorder). The authors explore the interaction between results of cerebral auditory-evoked response inhibition and maternal choline in relation to maternal infection [31] or marijuana use [33] during pregnancy. Freedman et al. [31] reported that choline levels at 16 weeks’ gestation were associated with lower (more normal) P50 amplitudes in 1-month-old infants whose mothers had experienced an infection, but there was no association among mothers who did not experience infection during pregnancy. The other report exploring P50 amplitudes also reported that lower (more normal) P50 amplitudes were positively associated with choline in infants whose mothers used marijuana during pregnancy [33]. Parent-reported behavior on the IBQ-R at 3 months of age showed a positive association between choline and increased regulation, but not surgency or negativity, only for infants whose mothers reported any kind of infection [31]. Further exploration of the IBQ results among the subgroup of mothers who experienced a viral respiratory infection showed a positive association between choline status ≥7.5 mM and increased regulation scores [32]. The attention subscale was higher among infants whose mothers experienced a viral respiratory infection and had a choline status of ≥7.5 mM [32]. Other regulation subscales were not reported, and neither were the IBQ-R surgency or negativity scores [32]. Likewise, choline was associated with better IBQ-R Regulation scores and many of the regulation subscales (although not surgency or negativity) for children whose mothers used marijuana [33]. In later assessments of these children, maternal choline concentrations >7.07 mM were associated with lower attention and problem scores, as well as fewer sleep problems in males only [34]. There were also interaction effects reported, where better attention scores were associated with higher maternal choline status among children whose mothers used cannabis compared to mothers who had not used cannabis, and among children whose mothers experienced an infection in pregnancy versus mothers who did not experience an infection [34]. Importantly, in this series of studies, authors claim that choline protects against the negative effects of infection [31,32,34] and marijuana use [33,34], and did not find associations with choline among mother–child pairs who did not experience infection [31,32].

## 4. Discussion

Current evidence is insufficient to support or refute the hypothesis that increasing choline intake in pregnancy improves child neurodevelopment. Although a meta-analysis was planned, the heterogeneity between the studies in terms of exposure or intervention, assessment used, sample characteristics (i.e., healthy samples versus heavy prenatal alcohol users), neurodevelopmental domain assessed, and age at assessment precluded an appropriate analysis of either the observational studies or the trials. The conclusions of both trials and observational studies somewhat differed, with some apparent bias and focus on reporting results with a positive association or group difference rather than the multitude of null findings that were present.

Most neurodevelopmental outcomes explored in the observational studies showed no association with prenatal choline. Nor were there consistent group differences among numerous outcomes compared in trials of choline supplementation. Although most observational studies used well-validated, age-standardized tests of neurodevelopment, there were different exposures at different time points in pregnancy. Four observational studies [11,29,31,32,33] measured circulating choline as an exposure indicator, which has limited accuracy and is considered a poor biomarker of choline intake. Whilst all observational studies adjusted for various confounders, the confounders selected were not consistent, and multiple important potential confounders were not considered. Limitations preventing confidence in trial results stem from the lack of a placebo group in one instance [27], samples being too small (ranging from 13 to 70 per group at enrolment) to confer power to detect a realistic effect on neurodevelopment and most suffering high attrition at the neurodevelopmental evaluation, which may introduce bias. Furthermore, most trial outcome assessments were experimental with subjective interpretation of performance. Results of the prenatal trial of choline with multivitamins excluded from this review would not have altered our conclusion as results were mixed (no benefit was detected on cognition or motor abilities at 6 months, although a small benefit for visual encoding and memory at 6–12 months) and subject to confounding due to no blinding, multiple randomization group, and the focus on the effect of alcohol intake [23,24].

Although we were able to include several new studies, our findings support the conclusions of previous reviews, that there are some suggestions of a benefit from increased prenatal choline for neurodevelopment, but mainly null effects and insufficient quality evidence to allow confidence in results [3,8,13,14,15,16,17,18]. One previous meta-analysis of prenatal choline exposure and offspring outcomes found an increased risk of neural tube defects with lower levels of circulating choline; however, it also did not deem it appropriate to attempt a meta-analysis of measures of child development [14]. The lack of strong or consistent benefits of prenatal choline in humans may be attributable to the body’s ability to synthesize the precursor to choline. Most pregnant women in populations consuming Western diets are not thought to be meeting their daily choline adequate intake; however, endogenous synthesis of the precursor may ensure a sufficient supply to adequately support neurodevelopment.

This review is limited by the quality of the available evidence base. The studies and trials to date are few and have limitations preventing confidence in the results, and the substantial variability in methodology prevents meta-analyses. Further, most studies have been conducted in North America, limiting generalizability to other populations. To ensure a comprehensive overview of the state of the evidence, we included results of studies and trials in journal publications, as well as conference abstracts, where available. However, it is worth noting that abstracts are not peer-reviewed and have not been published in full, despite being available for >4 years at the time of this review [40,41].

To allow the field to progress, a high-quality prenatal trial with a choline intervention, with a robust assessment of child neurodevelopment is needed. Such a trial needs to include a sufficient sample to detect a realistic effect of choline on neurodevelopment and must have an appropriate control group. Ideally, a new trial should be conducted in a population outside of North America with a naturally low choline intake.

## 5. Conclusions

Current evidence is insufficient to support or refute the hypothesis that increasing choline intake in pregnancy improves the neurodevelopmental outcomes of the child. A high-quality trial of choline supplementation during pregnancy with robust neurodevelopmental assessment beyond early childhood is needed to determine the optimal dose to support development.

## Figures and Tables

**Table 3:** Summary of risk of bias in clinical trials of prenatal choline supplementation and child neurodevelopment ^1^.

Author	Random Sequence Generation (Selection Bias)	Allocation Concealment (Selection Bias)	Blinding of Participants (Performance Bias)	Blinding of Outcome Assessment (Detection Bias)	Incomplete Outcome Data (Attrition Bias)	Selective Reporting (Reporting Bias)	Other Bias
Cheatham et al., 2012 [25]	+	+	+	?	+	−	?
Ross et al., 2013 [26]	−	?	?	?	−	?	?
Caudill et al., 2018 [27]	+	+	+	?	−	−	?
Jacobson et al., 2018 [28]	+	+	+	?	+	−	?

^1^+, Adequate (low risk of bias); ?, unclear (unknown risk of bias); −, inadequate (high risk of bias).

**Table 4:** Summary of quality of included cohort and case–control studies using the Newcastle–Ottawa Scale tool for evaluating the quality of non-randomized studies.

Author	Year	Selection				Comparability	Outcome			Total Score
Cohort Studies		Q1	Q2	Q3	Q4	Q1	Q1	Q2	Q3	
Signore et al. [29]	2008	*	*	*	*	**	*	*	-	8
Villamor et al. [30]	2021	*	*	-	*	**	-	*	*	7
Boeke et al. [12]	2013	*	*	-	*	**	-	*	-	6
Wu et al. [11]	2012	-	*	*	*	*	-	*	-	5
Hoffman et al. [33]	2020	*	*	*	*	*	*	*	*	8
Hunter et al. [42]	2021	*	*	*	*	*	*	*	-	7
Hunter et al. [35]	2021	*	*	*	*	**	*	*	-	7
Hunter et al. [34]	2022	*	*	*	*	*	*	*	-	8
Irvine et al. [36]	2023	*	*	-	*	*	*	*	-	6
		Selection				Comparability	Exposure			
Case Controls		Q1	Q2	Q3	Q4	Q1	Q1	Q2	Q3	
Freedman et al. [31]	2019	*	*	*	*	*	*	*	-	8
Freedman et al. [32]	2020	*	*	*	*	*	*	*	-	8

Newcastle-Ottawa scale for Cohort Studies: ‘Selection’ Q1, Representativeness of exposed cohort; Q2 Selection of non-exposed cohort; Q3, Ascertainment of exposure; Q4 Demonstration outcome of interest not present at the start of the study. ‘Comparability’ Q1, Comparability of cohorts on the basis of the design or analysis. ‘Outcomes’, Q1, Assessment of outcome; Q2, Follow-up long enough for outcomes to occur; Q3, Adequacy of follow-up of cohorts. A study can be awarded a maximum of one star for each numbered item within the Selection and Outcome categories. A maximum of two stars can be given for Comparability. Newcastle-Ottawa scale for Case control Studies: ‘Selection’ Q1, Is the case definition adequate; Q2, Representativeness of the cases; Q3, Selection of Controls; Q4, Definition of Controls. ‘Comparability’ Q1, Comparability of cases and controls on the basis of the design or analysis. ‘Exposure’ Q1, Ascertainment of exposure; Q2, Same method of ascertainment for cases and controls; Q3 Non-Response rate. A study can be awarded a maximum of one star (*) for each numbered item within the Selection and Exposure categories. A maximum of two stars (**) can be given for Comparability.

## Data Availability

No new data were created in the undertaking of this research.

## Abbreviations

The following abbreviations are used in this manuscript:

AI	Adequate Intake
Bayley	Bayley Scales of Infant Development
CBCL	Child Behavior Checklist
DCCS	Dimensional Change Card Sort
Fagan	Fagan Test of Infant Intelligence
FFQ	Food Frequency Questionnaire
K-ABC	Kaufman Assessment Battery
KBIT	Kaufman Brief Intelligence Test
MABC	Movement Assessment Battery for Children
MCDI	MacArthur-Bates Communicative Development Inventories
MCDI-SF	Mac- Arthur-Bates Short Form Vocabulary Checklist
MSEL	Mullen Scales of Early Learning
NEPSY	A Developmental Neuropsychological Assessment
NOS	Newcastle-Ottawa Scale
PPVT	The Peabody Picture Vocabulary Test
R	Revised
U.S.	United States (of America)
WPPSI	Wechsler Preschool and Primary Scale of Intelligence
WRAML	Wide Range Assessment of Memory and Learning
WRAVMA	Wide Range Assessment of Visual Motor Abilities

## References

[1] Zeisel S.H., da Costa K.A. (2009). Choline: An essential nutrient for public health. Nutr. Rev..

[2] Wiedeman A.M., Barr S.I., Green T.J., Xu Z., Innis S.M., Kitts D.D. (2018). Dietary Choline Intake: Current State of Knowledge Across the Life Cycle. Nutrients.

[3] Korsmo H.W., Jiang X., Caudill M.A. (2019). Choline: Exploring the Growing Science on Its Benefits for Moms and Babies. Nutrients.

[4] Australian Bureau of Statistics (2014). Australian Health Survey: Nutrition First Results—Foods and Nutrients.

[5] Probst Y., Sulistyoningrum D.C., Netting M.J., Gould J.F., Wood S., Makrides M., Best K.P., Green T.J. (2022). Estimated Choline Intakes and Dietary Sources of Choline in Pregnant Australian Women. Nutrients.

[6] Probst Y., Guan V., Neale E. (2019). Development of a Choline Database to Estimate Australian Population Intakes. Nutrients.

[7] Vennemann F.B., Ioannidou S., Valsta L.M., Dumas C., Ocké M.C., Mensink G.B.M., Lindtner O., Virtanen S.M., Tlustos C., D’addezio L. (2015). Dietary intake and food sources of choline in European populations. Br. J. Nutr..

[8] Derbyshire E., Obeid R. (2020). Choline, Neurological Development and Brain Function: A Systematic Review Focusing on the First 1000 Days. Nutrients.

[9] Bailey R.L., Pac S.G., Fulgoni V.L., Reidy K.C., Catalano P.M. (2019). Estimation of Total Usual Dietary Intakes of Pregnant Women in the United States. JAMA Netw. Open.

[10] Wallace T.C., Fulgoni V.L. (2016). Assessment of Total Choline Intakes in the United States. J. Am. Coll. Nutr..

[11] Wu B.T., Dyer R.A., King D.J., Richardson K.J., Innis S.M. (2012). Early second trimester maternal plasma choline and betaine are related to measures of early cognitive development in term infants. PLoS ONE.

[12] Boeke C.E., Gillman M.W., Hughes M.D., Rifas-Shiman S.L., Villamor E., Oken E. (2013). Choline intake during pregnancy and child cognition at age 7 years. Am. J. Epidemiol..

[13] Irvine N., England-Mason G., Field C.J., Dewey D., Aghajafari F. (2022). Prenatal Folate and Choline Levels and Brain and Cognitive Development in Children: A Critical Narrative Review. Nutrients.

[14] Obeid R., Derbyshire E., Schön C. (2022). Association between Maternal Choline, Fetal Brain Development, and Child Neurocognition: Systematic Review and Meta-Analysis of Human Studies. Adv. Nutr..

[15] Derbyshire E., Maes M. (2023). The Role of Choline in Neurodevelopmental Disorders-A Narrative Review Focusing on ASC, ADHD and Dyslexia. Nutrients.

[16] Jaiswal A., Dewani D., Reddy L.S., Patel A. (2023). Choline Supplementation in Pregnancy: Current Evidence and Implications. Cureus.

[17] Spoelstra S.K., Eijsink J.J.H., Hoenders H.J.R., Knegtering H. (2023). Maternal choline supplementation during pregnancy to promote mental health in offspring. Early Interv. Psychiatry.

[18] Serwatka C.A., Griebel-Thompson A.K., Eiden R.D., Kong K.L. (2023). Nutrient Supplementation during the Prenatal Period in Substance-Using Mothers: A Narrative Review of the Effects on Offspring Development. Nutrients.

[19] Zhong W., Hu L., Zhao Y., Li Z., Zhuo Y., Jiang X., Li J., Zhao X., Che L., Feng B. (2021). Effects of Dietary Choline Levels During Pregnancy on Reproductive Performance, Plasma Metabolome and Gut Microbiota of Sows. Front. Vet. Sci..

[20] Moher D., Hopewell S., Schulz K.F., Montori V., Gøtzsche P.C., Devereaux P.J., Elbourne D., Egger M., Altman D.G. (2010). CONSORT 2010 Explanation and Elaboration: Updated guidelines for reporting parallel group randomised trials. J. Clin. Epidemiol..

[21] Wells G.A., Shea B., O’Connell D., Peterson J., Welch V., Losos M., Tugwell P. (2021). The Newcastle-Ottawa Scale (NOS) for Assessing the Quality of Nonrandomised Studies in Meta-Analyses. https://www.ohri.ca/programs/clinical_epidemiology/oxford.asp.

[22] Moola S., Munn Z., Tufanaru C., Aromataris E., Sears K., Sfetcu R., Currie M., Qureshi R., Mattis P., Lisy K.M.P.F., Aromataris E., Munn Z. (2020). Chapter 7: Systematic reviews of etiology and risk. JBI Manual for Evidence Synthesis.

[23] Cifasd T., Coles C.D., Kable J.A., Keen C.L., Jones K.L., Wertelecki W., Granovska I.V., Pashtepa A.O., Chambers C.D. (2015). Dose and Timing of Prenatal Alcohol Exposure and Maternal Nutritional Supplements: Developmental Effects on 6-Month-Old Infants. Matern. Child. Health J..

[24] Kable J.A., Keen C., Uriu-Adams J., Jones K., Yevtushok L., Kulikovsky Y., Wertelecki W., Pedersen T., Chambers C. (2015). The impact of micronutrient supplementation in alcohol-exposed pregnancies on information processing skills in Ukrainian infants. Alcohol..

[25] Cheatham C.L., Goldman B.D., Fischer L.M., da Costa K.-A., Reznick J.S., Zeisel S.H. (2012). Phosphatidylcholine supplementation in pregnant women consuming moderate-choline diets does not enhance infant cognitive function: A randomized, double-blind, placebo-controlled trial. Am. J. Clin. Nutr..

[26] Ross R.G., Hunter S.K., McCarthy L., Beuler J., Hutchison A.K., Wagner B.D., Leonard S., Stevens K.E., Freedman R. (2013). Perinatal choline effects on neonatal pathophysiology related to later schizophrenia risk. Am. J. Psychiatry.

[27] Caudill M.A., Strupp B.J., Muscalu L., Nevins J.E.H., Canfield R.L. (2018). Maternal choline supplementation during the third trimester of pregnancy improves infant information processing speed: A randomized, double-blind, controlled feeding study. FASEB J..

[28] Jacobson S.W., Carter R.C., Molteno C.D., Stanton M.E., Herbert J.S., Lindinger N.M., Lewis C.E., Dodge N.C., Hoyme H.E., Zeisel S.H. (2018). Efficacy of Maternal Choline Supplementation During Pregnancy in Mitigating Adverse Effects of Prenatal Alcohol Exposure on Growth and Cognitive Function: A Randomized, Double-Blind, Placebo-Controlled Clinical Trial. Alcohol. Clin. Exp. Res..

[29] Signore C., Ueland P.M., Troendle J., Mills J.L. (2008). Choline concentrations in human maternal and cord blood and intelligence at 5 y of age. Am. J. Clin. Nutr..

[30] Villamor E., Rifas-Shiman S.L., Gillman M.W., Oken E. (2012). Maternal intake of methyl-donor nutrients and child cognition at 3 years of age. Paediatr. Perinat. Epidemiol..

[31] Freedman R., Hunter S.K., Law A.J., Wagner B.D., D’Alessandro A., Christians U., Noonan K., Wyrwa A., Hoffman M.C. (2019). Higher Gestational Choline Levels in Maternal Infection Are Protective for Infant Brain Development. J. Pediatr..

[32] Freedman R., Hunter S.K., Law A.J., D’Alessandro A., Noonan K., Wyrwa A., Hoffman M.C. (2020). Maternal choline and respiratory coronavirus effects on fetal brain development. J. Psychiatr. Res..

[33] Hoffman M.C., Hunter S.K., D’Alessandro A., Noonan K., Wyrwa A., Freedman R. (2020). Interaction of maternal choline levels and prenatal Marijuana’s effects on the offspring. Psychol. Med..

[34] Hunter S.K., Hoffman M.C., D’Alessandro A., Wyrwa A., Noonan K., Zeisel S.H., Law A.J., Freedman R. (2022). Prenatal choline, cannabis, and infection, and their association with offspring development of attention and social problems through 4 years of age. Psychol. Med..

[35] Hunter S.K., Hoffman M.C., D’Alessandro A., Walker V.K., Balser M., Noonan K., Law A.J., Freedman R. (2021). Maternal prenatal choline and inflammation effects on 4-year-olds’ performance on the Wechsler Preschool and Primary Scale of Intelligence-IV. J. Psychiatr. Res..

[36] Irvine N., England-Mason G., Field C.J., Letourneau N., Bell R.C., Giesbrecht G.F., Kinniburgh D.W., MacDonald A.M., Martin J.W., Dewey D. (2023). Associations between maternal folate status and choline intake during pregnancy and neurodevelopment at 3-4 years of age in the Alberta Pregnancy Outcomes and Nutrition (APrON) study. J. Dev. Orig. Health Dis..

[37] Ross R.G., Hunter S.K., Hoffman M.C., McCarthy L., Chambers B.M., Law A.J., Leonard S., Zerbe G.O., Freedman R. (2016). Perinatal Phosphatidylcholine Supplementation and Early Childhood Behavior Problems: Evidence for CHRNA7 Moderation. Am. J. Psychiatry.

[38] Bahnfleth C.L., Strupp B.J., Caudill M.A., Canfield R.L. (2022). Prenatal choline supplementation improves child sustained attention: A 7-year follow-up of a randomized controlled feeding trial. FASEB J..

[39] Warton F.L., Molteno C.D., Warton C.M.R., Wintermark P., Lindinger N.M., Dodge N.C., Zöllei L., van der Kouwe A.J., Carter R.C., Jacobson J.L. (2021). Maternal choline supplementation mitigates alcohol exposure effects on neonatal brain volumes. Alcohol. Clin. Exp. Res..

[40] Caudill M.A., Strupp B.J., Muscalu L., Nevins J.E., Canfield R.L. (2018). Maternal choline supplementation during pregnancy improves executive functioning in children at age 7 y (E10–06). Curr. Dev. Nutr..

[41] Bahnfleth C., Canfield R., Nevins J., Caudill M., Strupp B. (2019). Prenatal choline supplementation improves child color- location memory task performance at 7 y of age (FS05-01-19). Curr. Dev. Nutr..

[42] Hunter S.K., Hoffman M.C., McCarthy L., D’alessandro A., Wyrwa A., Noonan K., Christians U., Nakimuli-Mpungu E., Zeisel S.H., Law A.J. (2021). Black American Maternal Prenatal Choline, Offspring Gestational Age at Birth, and Developmental Predisposition to Mental Illness. Schizophr. Bull..

[43] Thomas J.D., Tran T.D. (2012). Choline supplementation mitigates trace, but not delay, eyeblink conditioning deficits in rats exposed to alcohol during development. Hippocampus.

